# Evaluation of CoVid-19 infection among vaccinated and unvaccinated individuals using biochemical markers

**DOI:** 10.6026/973206300200223

**Published:** 2024-03-31

**Authors:** Allavarapu Ramya Sree, K Sethumadhavan, S.P. Tejaswi Pullakanam, Purimitla Usharani

**Affiliations:** 1 Department of Microbiology, Aarupadai Veedu Medical College and Hospital, Vinayaka Mission's Research Foundation (VMRF-DU), Puducherry, Tamil Nadu, India; 2 Department of Biochemistry, Gayatri Vidya Parishad Institute of Health Care and Medical Technology, Marikavalasa, Visakhapatnam, Andhra Pradesh, India; 3 Department of Microbiology, Dr. Patnam Mahender Reddy Institute of Medical Sciences, Chevella, Ranga Reddy District, Telangana, India

**Keywords:** CoVid-19, vaccinated, unvaccinated, symptoms, prevalence, ferritin, D-dimer, CRP

## Abstract

The evaluation of infection rate for CoVid-19 cases among vaccinated and unvaccinated individuals using haematological parameters is of interest. Data such as age, gender, occupation, location, signs and symptoms, length of symptoms, date of sample collection and report generation, status of vaccinations, and outcome available in the database was used in this analysis. Ferritin levels, D-dimer values, CRP, troponin-1 levels and platelet count of each CoVid-19 patient were recorded and analysed. Data shows that maximum number of cases was reported during the second wave 143 (51.07%). The common age group affected was 31-40 years 28.56%. The common symptom identified was weakness in arms and legs among the unvaccinated group of CoVid-19 positive group. However, common symptom identified was fatigue (87%) among those who received only the first dose of CoVid vaccine. Nonetheless, the symptom identified was hair loss (68%) among those who received both the doses of CoVid vaccine. Thus, data shows that people do experience severe and life-threatening COVID-19 infection despite receiving the vaccination. Nonetheless, the infection is mild and very few individuals require hospitalization. No deaths are reported in the group that received the vaccination. This is in contrast to the unvaccinated group, which had a severe CoVid-19 infection with few deaths reported.

## Background:

Coronavirus disease 2019 (COVID-19) is a highly contagious viral infection brought on by the SARS-CoV-2 coronavirus, which causes severe acute respiratory syndrome. More than 6 million people have died as a result of the terrible effects of COVID-19 [1]. According to WHO, there are several COVID-19 vaccines validated for use. The first mass vaccination programme started in early December 2020. The vaccines listed as Pfizer/Biotech Comirnaty vaccine (manufactured in December 2020), SII/COVISHIELD and AstraZeneca/AZD1222 vaccines (manufactured in February 2021), Janssen/Ad26.COV 2.S vaccine (developed by Johnson & Johnson, March 2021), Moderna COVID-19 vaccine (mRNA 1273) (developed on April 2021), Sinopharm COVID-19 vaccine (developed on May 2021), Sinovac-CoronaVac vaccine (manufactured by June 2021), Bharat Biotech BBV152 COVAXIN vaccine (developed on November 2021), Covovax (NVX-CoV2373) vaccine (developed on December 2021) and Nuvaxovid (NVX-CoV2373) vaccine (manufactured on December 2021) [2]. All vaccines are given in two doses except Janssen/Ad26.COV 2.S vaccine given in one dose and Zydus Cadila's DNA vaccine (ZyCov-D) manufactured in Aug 2021 given in 3 doses [3]. Among these vaccines Covishield (ChAdox1 nCov-19) and Covaxin (BBV152) were widely used in the mass vaccination programme in India. A break through Infection was defined as the detection of SARS-Cov-2 on RT-PCR assay performed 11 or more days after receipt of a second dose of CoVid-19 vaccine and if no explicit exposure or symptoms had been reported during the first 6 days. It is difficult to achieve sterilizing immunity with SARs-Cov-2 vaccination and protection is going to be reduced even upon vaccination with time [4]. Therefore the main research questions for the researchers include studying CoVid-19 infections are- surrounded their timing, frequency, causes, severity and levels of Infectiousness. The answers to these questions matter for several reasons. First, identifying the frequency, severity and causes of infections may inform the choice of public health responses: watchful waiting may be appropriate of are markedly increase transmission rates. Identifying the range of clinical outcomes seen in infections and determining how severe they can be as well as which clinical and demographic individual characteristics are associated with a severe outcome, will indicate how information about vaccination history can be used in prognostic scores to identify who should receive priority for additional vaccination or treatments [5]. To find out the occurrence of infections, it is important to compare the incidence rate of infections to the rate of non- infections in unvaccinated people who apart from their vaccination status, are similar to the vaccinated [6]. This comparison provides an estimate of vaccine effectiveness. These vaccines have also been shown to reduce the incidence of asymptomatic infection and the associated infectivity. However, break through infections have emerged in some vaccine recipients. To that no correlate of protection from break through infection has been reported. Unpublished data from the open-label phase III clinical trial of the pfizer and modern mRNA vaccines compares infections during a period in July and August 2021 among individuals randomized to vaccination at the start of the trail versus those originally randomized to placebo who received the vaccine later, following unblinding. In each cases were more frequently in the earlier vaccination individuals, providing randomized evidence for waning vaccine efficacy. When taking into account of prior studies, the utilization of circulating biomarkers for the immune system and inflammation has been thought of as a prognostic indication in COVID-19-positive individuals. However, their diagnostic value has not been well investigated, and there is no information on the influence of haematological markers on CoVid-19 infection [7]. Therefore, it is of interest to determine the infection rate among the CoVid-19 cases admitted in a tertiary care teaching hospital. We also compared the incidence of CoVid-19 infection among the vaccinated and unvaccinated individuals. We further compared the influence of haematological parameters in the individuals with infection with that of unvaccinated individuals.

## Methodology:

## Settings:

It is a retrospective study conducted on 381 patients, both IP and OP, who presented with COVID-19 symptoms in a Tertiary Care hospital, Telangana out of 1000 reported cases. Informed consent was taken from each individual before the collection of swabs. The study was undertaken after obtaining approval from the Institutional Ethics Committee.

## Study design, sampling and methodology:

All confirmed infections of SARS-COV-2 were recorded in the daily CoVid-19 confirmed cases database of PCR laboratory. This database contains individual level data of infected patient like age, gender, occupation, locality, signs and symptoms, duration of symptoms, date of sample collection and report generated results, vaccination status, outcome etc. We conducted a two years and 1 month longitudinal retrospective observational study starting from Oct 2020 to Nov 2022. It concerned all the CoVid-19 positive cases and both vaccinated (fully or partially vaccinated) and unvaccinated individuals over the study period. The inclusion criteria were all the fully, partially vaccinated and unvaccinated patients who tested positive for SARS-CoV-2 infection and those who tested negative for Sars-CoV2 were excluded in the study. Children under ten years old and patients unwilling to consent were also excluded from the study. Individuals with mental or psychiatric severe issues and those with active tuberculosis were not allowed to participate in the study. Further the CoVid-19 positive samples were screened based on the variants.

Throughout the hospitalization period, the ferritin levels of each CoVid-19 patient were noted. According to the manufacturer's instructions, serum ferritin was tested using the COBAS 6000 from Roche Diagnostics, Indianapolis, IN, USA with Chemiluminescence immunoassay (CLIA) method. Using a conventional F200 analyser, with immunofluorescence method used to measure D-dimer levels. Serum troponin I (TnI) levels were determined using a chemiluminescent immunoassay with an ADVIA Centaur XP system from Siemens Medical Solutions in Malvern, Pennsylvania, and serum CRP levels were determined based on the principle of latex agglutination. CBC SYSMEX XK -21N haematology analyser was used to analyse the platelet count using EDTA samples taken from the patients' peripheral venepunctures. The platelet trend was also examined.Data were entered into Microsoft Office 2019 Excel sheets (Microsoft® Corp., Redmond, USA) and analyzed using Statistical Package for Social Sciences (SPSS) version 24.0 (IBM Corp., NY, USA), The data were presented as percentages, means, and standard deviation.

## Results:

Table 1 shows the age-wise distribution of vaccinated positive CoVid-19 cases. The results revealed that maximum number of cases was reported from age group 31-40 years 28.57% and least number of cases was reported from <20 age group 1.78% among the 280 total cases of vaccinated CoVid-19 positive group. Table 2 represents the age-wise distribution of unvaccinated positive CoVid-19 cases. The results revealed that maximum number of cases was reported from age group 41-50 years 28.71% and least number of cases was reported from >60 age group 4.95% among the 101 total cases of unvaccinated CoVid-19 positive group. Table 3 showed the results of CoVid Wave wise distribution of total vaccinated CoVid-19 positive cases (280) with respect to age. Among the data for three waves was compared the maximum number of cases were reported during the second wave 143 (51.07%). The common age group affected was 31-40 years 28.56%. Table 4 shows the results of CoVid Wave wise distribution of total unvaccinated CoVid-19 positive cases (101) with respect to age. Among the data for three waves was compared the maximum number of cases were reported during the second wave 48 (47.52%). The common age group affected was 41-50 years 28.7%. Among the vaccinated group (61.43%) and unvaccinated groups (58.42%), male prevalence was high in CoVid-19 positive cases (Table 5). Among the vaccinated group (61.78%) and unvaccinated groups (68.31%), more CoVid-19 positive cases (Table 6) were reported from rural areas. Table 7 depicted the symptoms wise distribution of unvaccinated, 1st dose, 2nd dose positive CoVid-19 cases. The most common symptom identified was weakness in arms and legs among the unvaccinated group of CoVid-19 positive group. Where as in those patients who received only first dose of CoVid vaccine, and the most common symptom identified was fatigue (87%) and those who received both the doses of CoVid vaccine, the most common symptom identified was hair loss (68%). Table 8 depicted the influence of haematological parameters in CoVid-19 infected vaccinated and unvaccinated groups. The mean value of ferritin levels in unvaccinated CoVid-19 infected group (215.27±136.3) was higher than vaccinated group (72.94±123.2). The mean value of D-dimer in unvaccinated CoVid-19 infected group (4.71±25.71) was higher than vaccinated group (0.67±3.41). The mean value of troponin-1 levels in unvaccinated CoVid-19 infected group (0.21±0.437) was higher than vaccinated group (0.07±0.28). The mean value of CRP levels in unvaccinated CoVid-19 infected group (23.84±16.06) was higher than vaccinated group (8.62±15.49). The mean value of Platelet levels in unvaccinated CoVid-19 infected group (4.34±6.6) was higher than vaccinated group (1.51±4.32). The Pearson correlation results revealed all the haematological parameters were negatively, insignificantly correlated in both vaccinated and unvaccinated groups. When paired t-test analysis was performed all the haematological variables were significantly influenced in both vaccinated and unvaccinated groups.

## Discussion:

It is difficult to achieve sterilizing immunity with SARs-Cov-2 vaccination and protection is going to be reduced even upon vaccination with time. Therefore, timing, frequency, causes, severity and levels of infection are key parameters. Maximum number of cases was reported from age group 31-40 years 28.57% among the 280 total cases of vaccinated CoVid-19 positive group whereas maximum number of cases was reported from age group 41-50 years 28.71% from 101 total cases of unvaccinated CoVid-19 positive group. Among the vaccinated group (61.43%) and unvaccinated groups (58.42%), male prevalence was high in CoVid-19 positive cases. Similar reports were revealed by Shahapur *et al.* 2022 [8]. The mean age of study participants was 46.22±17.77 years. Of the patients included, 64 (62.1%) were males and 39 (37.9%) were females. Lee *et al.* 2022 [9] found that, among the 761 hospitalized patients with COVID-19, the mean age was 47 years, 47 patients (6%) were fully vaccinated (infection), 127 (17%) were partially vaccinated, and 587 (77%) were unvaccinated. Where as in the present the number of fully vaccinated individuals were 138 (36.2%), partially vaccinated individuals 142 (37.2%) and 101 were unvaccinated individuals (26.5%) among the 381 CoVid-19 infected reported cases. The maximum number of reported cases of CoVid-19 infection were from partially vaccinated group 50.7% (took only first dose of vaccine) than fully vaccinated group 49.3% among the 280 vaccinated individuals. Similar findings were reported with Ali *et al.* 2022 [10]; Amit *et al.* 2021 [11]; Keehner *et al.* 2021 [12]; Sabnis *et al.* 2021 [13] and Kandeel *et al.* [14]. Shahapur *et al.* 2022 [8] reported the most of the symptomatic patients of infection were suffered with episodes of fever, headache, sore throat, fatigue, cough, and breathlessness. In the present study the most common symptom identified was weakness in arms and legs among the unvaccinated group of CoVid-19 positive group. Where as in those patients who received only first dose of CoVid vaccine, and the most common symptom identified was fatigue (87%) and those who received both the doses of CoVid vaccine, the most common symptom identified was hair loss (68%). Most of the CoVid-19 infected individuals were reported with increased serum ferritin levels. In the current research, the mean value of ferritin levels in unvaccinated CoVid-19 infected group (215.27±136.3) was higher than vaccinated group (72.94±123.2). The results were in accordance to Korishettar *et al.* 2022[15] found that the mean serum ferritin level was significantly higher in unvaccinated (665.85 ± 557.70 ng/mL) patients by more than two-fold compared to vaccinated patients. Strong correlation of serum ferritin levels and CoVid-19 infection were reported by many previous research studies [16,17,18]. The mean value of D-dimer in unvaccinated CoVid-19 infected group (4.71±25.71) was higher than vaccinated group (0.67±3.41). The mean value of troponin-1 levels in unvaccinated CoVid-19 infected group (0.21±0.437) was higher than vaccinated group (0.07±0.28).The mean value of CRP levels in unvaccinated CoVid-19 infected group (23.84±16.06) was higher than vaccinated group (8.62±15.49). Fatima *et al.* 2022 [19] reported the similar findings that, the mean values of D-dimer and serum troponin-1 were higher for unvaccinated individuals (1.2, 0.25) than fully vaccinated individuals (0.8, 0.05). Trofin *et al.* 2023 [20] reported the higher mean values of CRP levels in vaccinated individuals of CoVid-19 infection. The mean value of Platelet levels in unvaccinated CoVid-19 infected group (4.34±6.6) was higher than vaccinated group (1.51±4.32) and these results agreed with the findings of Ostrowski *et al.* 2021 [21]. Fully vaccinated patients had significantly lower mean hospital days and case fatality rate than the unvaccinated and partially vaccinated patients when the effectiveness of the CoVid-19 vaccine for the prevention of severe disease and death during hospitalization was evaluated. Similar findings were reported by Kandeel *et al.* 2023 [14], Ali *et al.* 2022 [22] and Shahapur PR *et al.* [23].

## Conclusion:

Patients develop severe and critical COVID-19 infection despite being vaccinated. But in most of the cases the infection is mild and very few patients were hospitalized. Mortality was reported in the vaccinated group. However, CoVid-19 infection was severe and few deaths were reported in the unvaccinated group.

## Source(s) of support:

Nil

## Figures and Tables

**Table 1:** Age-wise distribution of vaccinated positive CoVid-19 cases

**Vaccinated (280)**	**Count**	**%**
<20	5	1.78
21-30	26	9.28
31-40	80	28.57
41-50	74	26.42
51-60	63	22.5
>60	32	11.4
Total	280	

**Table 2:** Age-wise distribution of unvaccinated positive CoVid-19 cases

**Unvaccinated (101)**	**Count**	**%**
<20	8	7.92
21-30	15	14.85
31-40	28	27.72
41-50	29	28.71
51-60	16	15.84
>60	5	4.95
Total	101	

**Table 3:** CoVid wave wise distribution of vaccinated CoVid-19 positive cases with respect to age

	**Count (5) Age <20**	**%**	**Count (26) Age 21-30**	**%**	**Count (80) Age 31-40**	**%**	**Count(74) Age 41-50**	**%**	**Count (63) Age 51-60**		**Count(32) Age >60**	**%**	**Total**
First wave	2	0.71	8	2.85	29	10.35	23	8.21	19	6.78	10	5.57	91
Second wave	2	0.71	14	5	37	13.21	39	13.92	34	12.14	17	6.07	143
Third wave	1	0.35	4	1.42	14	5	12	4.28	10	5.57	5	1.78	46
Total	5	1.77	26	9.27	80	28.56	74	26.41	63	24.49	32	13.42	280

**Table 4:** CoVid Wave wise distribution of unvaccinated CoVid-19 positive cases with respect to age

	**Count (8) Age <20**	**%**	**Count (15) Age 21-30**	**%**	**Count (28) Age 31-40**	**%**	**Count(29) Age 41-50**	**%**	**Count (16) Age 51-60**	**%**	**Count(5) Age >60**	**%**	**Total**
First wave	2	1.98	4	3.96	7	6.93	9	8.91	5	4.95	1	0.99	28
Second wave	5	4.95	8	7.92	12	11.88	13	12.87	7	6.93	3	2.97	48
Third wave	1	0.99	3	2.97	9	8.91	7	6.93	4	3.96	1	0.99	25
Total	8	7.92	15	14.85	28	27.72	29	28.71	16	15.84	5	4.95	101

**Table 5:** Sex-wise distribution of vaccinated and unvaccinated positive CoVid-19 cases

**Vaccinated group**	**Female count**	**%**	**Male count**	**%**	**Total**	**%**
	108	38.57	172	61.43	280	100
Unvaccinated group	Female count	%	Male count	%	Count	%
	42	41.58	59	58.42	101	100

**Table 6:** Residency wise distribution of vaccinated and unvaccinated positive CoVid-19 cases

	**Rural count**	**%**	**Urban count**	**%**	**Total count**
Vaccinated group	173	61.78%	107	38.21%	280
Unvaccinated group	69	68.31%	32	31.68%	101

**Table 7:** Symptoms wise distribution of unvaccinated, 1st dose, 2nd dose positive CoVid-19 cases

			**Vaccinated (n=280)**			
Post CoVid symptoms (total)	Unvaccinated (101)	%	1st dose of vaccine (n=142) 50.7%	%	2nd dose of vaccine (n=138) 49.3%	%
Fatigue (271)	91	90.10%	124	87%	56	41%
Dyspnea(192)	74	73.26%	95	67%	23	16%
Depression, anxiety and stress (264)	85	84.15%	99	70%	80	58%
Hair loss (278)	83	82%	101	71%	94	68%
Insomnia(102)	39	38.61%	53	37%	10	7%
Weakness in arms and legs (285)	98	97%	122	86%	65	47%

**Table 8:** Influence of haematological parameters in CoVid-19 infected individuals of both vaccinated and unvaccinated group

**Haematological variables**		**Mean**	**Std. Deviation**	**Std. Error Mean**	**Pearson correlation**	**Significant**	**'t' value**	**Sig. (2-tailed)**
Ferritin	Unvaccinated	215.27	136.36	8.149	-0.054	0.371	12.623	0
	Vaccinated	72.94	123.28	7.367				
D-dimer	Unvaccinated	4.71	25.71	1.536	-0.03	0.623	2.594	0.01
	Vaccinated	0.67	3.413	0.203				
Troponin - 1	Unvaccinated	0.21	0.437	0.026	-0.046	0.438	4.36	0
	Vaccinated	0.07	0.286	0.017				
CRP	Unvaccinated	23.84	16.06	0.96	-0.007	0.912	11.374	0
	Vaccinated	8.62	15.49	0.925				
Platelet count	Unvaccinated	4.34	6.6	0.394	-0.037	0.541	5.901	0
	Vaccinated	1.51	4.32	0.258				

**Table 9:** Hospitalization and mortality wise distribution of vaccinated and unvaccinated positive CoVid-19 cases

	**Hospitalized**	**%**	**Non-hospitalized**	**%**	**Death**	**%**
Vaccinated group	8	2.85	272	97.15	0	0
Unvaccinated group	22	21.78	79	78.22	2	1.98
